# Oral administration of *Bifidobacterium bifidum* G9-1 alleviates rotavirus gastroenteritis through regulation of intestinal homeostasis by inducing mucosal protective factors

**DOI:** 10.1371/journal.pone.0173979

**Published:** 2017-03-27

**Authors:** Tomohiro Kawahara, Yutaka Makizaki, Yosuke Oikawa, Yoshiki Tanaka, Ayako Maeda, Masaki Shimakawa, Satoshi Komoto, Kyoko Moriguchi, Hiroshi Ohno, Koki Taniguchi

**Affiliations:** 1 R&D Center, Biofermin Pharmaceutical Co., Ltd., Kobe, Japan; 2 Department of Virology and Parasitology, Fujita Health University School of Medicine, Toyoake, Aichi, Japan; University of Palermo, ITALY

## Abstract

Human rotavirus (RV) infection is a leading cause of dehydrating diarrhea in infants and young children worldwide. Since therapeutic approaches to RV gastroenteritis are limited to alleviation of dehydration with oral rehydration solutions, more direct approaches to palliate symptoms of RV gastroenteritis are required. Treatments with probiotics have been increasingly recognized as alternative safe and low cost treatments for moderate infectious diarrhea. In this study, *Bifidobacterium bifidum* G9-1 (BBG9-1), which has been used as an intestinal drug for several decades, was shown to have a remarkable protective effect against RV gastroenteritis in a suckling mice model. As well as prophylactic oral administration of BBG9-1 from 2 days before RV infection, therapeutic oral administration of BBG9-1 from 1 day after RV infection significantly alleviated RV-induced diarrhea. Therapeutic administration of BBG9-1 reduced various types of damage in the small intestine, such as epithelial vacuolization and villous shortening, and significantly diminished the infectious RV titer in mixtures of cecal contents and feces. It was also shown that therapeutic administration of BBG9-1 significantly increased the number of acidic mucin-positive goblet cells and the gene expression of mucosal protective factors including MUC2, MUC3, MUC4, TGFβ1 and TFF3 in the small intestine. This led to alleviation of low gut permeability shown as decreased gene expression levels of occludin, claudin-1 and villin-1 after RV infection. Furthermore, in the small intestine, therapeutic administration of BBG9-1 significantly palliated the decreased gene expression of SGLT-1, which plays an important role in water absorption. In the large intestine, administered BBG9-1 was shown to replicate to assimilate undigested nutrients, resulting in normalization of the abnormally high osmotic pressure. These results suggested that water malabsorption caused by RV infection was alleviated in mice administered BBG9-1. Thus, the present study showed that oral administration of BBG9-1 palliated diarrhea partly through protection against RV-induced lesions by inducing mucosal protective factors. Oral administration of BBG9-1 is thought to be an efficient method for management of an RV epidemic for both prophylactic and therapeutic purposes.

## Introduction

Human rotavirus (RV) infection is a leading cause of dehydrating infantile gastroenteritis worldwide. The symptoms of RV gastroenteritis are vomiting, watery diarrhea, dehydration, and low-grade fever. It has been reported that RV causes symptoms beyond the gastrointestinal, including neurological manifestations such as encephalopathy, though infrequently. Most symptomatic episodes occur in young children between the ages of 3 months and 2 years[1]. The high transmissibility of RV, the infective doses of which are presumed to be 10–100 infectious viral particles, also highlights RV gastroenteritis as one of the most noteworthy infectious diseases, especially in young children. RV is transmitted by a fecal-oral route, and the virus infects and damages mature enterocytes in the small intestine, resulting in malabsorption of Na^+^ and water and a decrease in digestive enzymes such as lactase. Transition of undigested nutrients including disaccharides into the colon shifts the osmotic pressure gradient in a positive direction, resulting in inhibition of absorption of sufficient water in the colon. Along with malabsorption of water, increased chloride secretion and shortened intestinal transit time, which are largely evoked by virus-encoded nonstructural protein 4 (NSP4) as an enterotoxin, have also been reported to be involved in RV-induced diarrhea[2,3].

For preventive measures against RV gastroenteritis, two RV vaccines have been licensed in more than 100 countries worldwide since 2006. Several studies have demonstrated their safety and efficacy in both reducing the incidence of RV infection and alleviating symptoms[4,5]. However, lower performance of RV vaccines has been reported in developing countries, and concurrent enteric infections and malnutrition have been pointed out as major reasons. Therapeutic approaches to RV gastroenteritis include the administration of an oral rehydration solution that reduces dehydration after RV infection. However, this treatment does not alleviate diarrhea itself[6,7]. Thus, an alternative treatment for RV gastroenteritis has been required.

Probiotics are defined as live microorganisms that confer a health benefit on the host when administered in adequate amounts[8]. Some probiotics, as represented by lactic acid bacteria, have been used to improve abdominal symptoms resulting from changes in the intestinal microbiota. Recently, probiotics have been increasingly recognized as alternative safe and low cost treatments for moderate infectious diarrhea[7,9,10]. *Bifidobacterium bifidum* is one of the major intestinal microflora components in children, and its symbiotic associations have been reported: mammalian lactation promotes intestinal colonization of *Bifidobacterium bifidum*, which in turn protects and guides the intestinal health and immune system in infants[11]. *Bifidobacterium bifidum* G9-1 (BBG9-1) was isolated from a healthy person, and its high level of safety is certified by the fact that BBG9-1 has been used as an intestinal drug for several decades. In addition to its anti-flatulent effect, other health promotion effects of BBG9-1, including regulation of allergic reactions[12,13], hypertriglyceridemia and hyperglycemia[14], have been investigated.

Several studies have shown the efficacy of probiotics against RV gastroenteritis in animal models, but in most studies, probiotics were used for prophylactic purposes by administrating probiotics before RV infection[15,16,17,18,19]. Recently, Zhang *et al*. showed that both pre-treatment and post-treatment with *Lactobacillus rhamnosus* GG alleviates RV-induced diarrhea through induction of mucosal IgA and sIgA[20]. In contrast to prophylactic approaches, little is known about the effects and underlying mechanisms of therapeutic administration of probiotics for RV gastroenteritis, especially in a stage where diarrhea is observed.

We report here that both prophylactic and therapeutic oral administrations of BBG9-1 alleviate RV gastroenteritis including diarrhea and intestinal lesions through protection of intestinal epithelia by induction of mucosal protective factors in a suckling mouse model.

## Materials and methods

### Bacteria

BBG9-1 was obtained from the Culture Collection of Biofermin R&D Center. BBG9-1 was cultured for 18 h at 37°C in GAM broth (Nissui Pharmaceutical Co., Ltd., Tokyo, Japan) supplemented with 0.7% glucose and 0.1% Tween-80. The bacteria were washed twice with phosphate buffered saline (PBS), and the pellets obtained after low-speed centrifugation were stored at -80°C until use.

### Cells

Fetal rhesus monkey kidney MA-104[21] cells and African green monkey kidney CV-1[21] cells were grown in Eagle’s minimum essential medium (E-MEM, Sigma-Aldrich, St Louis, MO, USA) supplemented with 5% heat-inactivated fetal bovine serum (FBS) (Gibco Life Technologies, Gaithersburg, MD, USA) and gentamycin (50 μg/ml) (Gibco). MA-104 cells and CV-1 cells were incubated at 37°C under a humidified atmosphere of 5% CO_2_ in air. MA-104 cells were used for propagation of the simian rotavirus strain SA11. CV-1 cells were used for measurement of virus titers in mixtures of cecum and colonic contents.

### Virus

Strain SA11[21] was activated by trypsin from a porcine pancreas (p-trypsin, 10 μg/ml at a final concentration) (Sigma-Aldrich) for 30 min at 37°C. After washing with PBS containing calcium and magnesium (Sigma-Aldrich), MA-104 cells were inoculated with activated SA11 virus and incubated for 30 min at 37°C. The infected MA-104 cells were cultured in FBS-free MEM containing p-trypsin (1 μg/ml) for 3 days at 37°C and freeze-thawed twice. The supernatant of cultured fluids was stored at -80°C until use.

### Animals

Pregnant BALB/c mice (14–18 days of gestation) were obtained from Japan SLC, Inc. (Hamamatsu, Japan). The mice were housed individually, and standard diet (CE-2 pellets; CLEA Japan, Inc., Tokyo, Japan) and water were given *ad libitum*. Pups were born on day 19 of gestation. This animal study was approved by the Experimental Animal Care and Use Committee of Biofermin Pharmaceutical Co., Ltd.

### RV infection model

Two-day-old suckling mice were randomly divided into a control group (RV-Control group) and a BBG9-1 group (RV-BBG9-1 group). For prophylactic administration of BBG9-1, suckling mice in the RV-Control group and those in the RV-BBG9-1 group were orally administered 50 μl of PBS and PBS containing 3.0 ×10^7^ colony-forming units (CFU) of BBG9-1, respectively, once daily for 10 days from 2 days before to 7 days after RV infection including the day of RV infection. For therapeutic administration of BBG9-1, suckling mice in the RV-Control group and those in the RV-BBG9-1 group were orally administered 50 μl of PBS and PBS containing 3.0 ×10^7^ CFU of BBG9-1, respectively, once daily for 7 days from 1 to 7 days after RV infection. Seven-day-old suckling mice were orally inoculated with 50 μl of cell-cultured supernatant containing 1.5 ×10^6^ plaque-forming units (PFU) of strain SA11. To determine the severity of diarrheal index, incidence rate of diarrhea and diarrhea score were measured daily until 8 days after RV infection. Incidence rate of diarrhea was calculated in litters of 3–7 pups/dam. Diarrhea score was measured as previously described[22,23,24] with some modifications. Briefly, diarrhea score was measured individually on the basis of the following scale: 0: normal; 1: loose stool; 2: moderate diarrhea (defined as orange-yellow colored stool); 3: severe diarrhea (defined as light yellow colored stool). For examination of the therapeutic effects of BBG9-1, suckling mice at 2 days post infection (dpi) were subjected to analyses of histopathology and gene expression of the small intestine, concentrations of acetic acid and L-lactic acid, and pH and microbiota in small intestinal content and mixtures of cecum and colonic contents. Furthermore, mixtures of cecum and colonic contents from suckling mice on days 2–5 were subjected to measurement of infectious RV titers by a plaque assay. In this model, clinical symptoms other than diarrhea were not observed during the infection experiments. All procedures for handling the infectious agent were conducted under biosafety level-2A conditions.

### Non-infection mouse model

Seven-day-old suckling mice were orally administered 50 μl of PBS instead of rotavirus suspension. Eight-day-old suckling mice were orally administered 50 μl of PBS. Nine-day-old suckling mice were subjected to analyses of histopathology and gene expression of the small intestine, concentrations of acetic acid and L-lactic acid, and pH and microbiota in small intestinal content and mixtures of cecum and colonic contents.

### Histopathology of the small intestine

Under anesthesia, the jejunum and ileum were harvested and fixed with Carnoy’s solution (Wako Pure Chemical Industries, Ltd., Osaka, Japan) for 3 h at 4°C, followed by substitution with 70% EtOH. Preparation of paraffin blocks, hematoxylin and eosin (HE) staining, and high iron diamine-alcian blue (HID/AB) staining were performed at Biopathology Institute Co., Ltd. (Oita, Japan). H&E-stained sections were used for analyses of RV-induced lesions. Histopathological scores in the jejunum and ileum were evaluated by the sum of the following three histopathological aspects of villi: epithelial vacuolization, assigned a grade of 0–3 to indicate whether there was no vacuolization (0) or whether there was vacuolization in one third (1), two thirds (2), or all (3) of the villi; breakage of the epithelial barrier, assigned a grade of 0 or 1 to indicate no loss (0) or loss of (1) of epithelial cells; villus disruption, assigned a grade 0 or 1 to indicate the absence (0) or presence (1) of a broken or damaged appearance of the villus[25]. For evaluation of villus shortening, villus lengths in the jejunum and ileum were measured by NIH Image J software and classified into four groups with lengths of less than 100 μm, between 100 and 150 μm, between 150 and 200 μm and more than 200 μm. In HID/AB-stained sections, HID-positive brown stain and AB-positive blue stain were defined as goblet cells containing sulfated mucins and sialomucins, respectively. The number of goblet cells containing each mucin was calculated per length of villi. The jejunum and ileum sections were observed using an AxioVert 200M optical microscope (Carl Zeiss, Göttingen, Germany).

### Virus titration in mixtures of cecum and colonic contents

For activation of RV, mixtures of cecum and colonic contents were incubated with E-MEM containing acetylated trypsin (30 μg/ml) (Sigma-Aldrich) for 30 min at 37°C. After washing with E-MEM, MA-104 cells were inoculated with 300 μl of mixtures of cecum and colonic contents serially diluted 10 times and incubated at 37.0°C for 1 h. After washing with FBS-free E-MEM, monolayers of the cells were overlaid with 2.5 ml of E-MEM supplemented with p-trypsin (2 μg/ml) and 0.7% SeaKem ME agarose (Lonza, Rockland, ME, USA) at 37°C for 48 h. MA-104 cells were then stained with 2 ml of E-MEM containing 0.7% SeaKem ME agarose and 0.005% neutral red (Sigma-Aldrich) at 37°C for 24 h. Virus titers (PFU/mg) were determined by counting plaques.

### Analysis of gene expression in the small intestine

The jejunum and ileum were finely minced and then immersed in RNAlater (Ambion Life Technologies, Carlsbad, CA, USA) and stored at -20°C until use. Total RNA was extracted using a Qiagen RNeasy Midi kit according to the manufacturer's protocol (Qiagen, Valencia, CA, USA). cDNA was synthesized using SuperScript® VILO™ MasterMix (Invitrogen Life Technologies). The mRNA levels of transforming growth factor (TGF) β1, trefoil factor family peptide 3 (TFF3), mucin-2 (MUC2), MUC3, MUC4, occludin, claudin-1, villin-1, lactase and sodium glucose cotransporter-1 (SGLT-1) were determined by real-time quantitative PCR using an ABI Prism 7000 SDS (Applied Biosystems Life Technologies) with the TaqMan® Gene expression assay (TGFβ1, Mm01178819_m1; TFF3, Mm00495590_m1; MUC2, Mm01276696_m1; MUC3, Mm01207064_m1; MUC4, Mm00466886_m1; occludin, Mm00500912_m1; claudin-1, Mm00516701_m1; villin-1, Mm00494146_m1; lactase, Mm01285112_m1; SGLT-1, Mm00451203_m1; Applied Biosystems Life Technologies). The mRNA level of glyceraldehyde-3-phosphate dehydrogenase (GAPDH) was measured using TaqMan® Rodent GAPDH Control Reagent (4308313, Applied Biosystems Life Technologies) as an endogenous control for normalizing each gene expression. The mRNA levels of genes in the RV-Control and RV-BBG9-1 groups are shown as relative values to those in non-infected mice (Non-RV group).

### Measurement of pH in contents of the small intestine and contents of the cecum and colon

Contents of the small intestine and contents of the cecum and colon were carefully suspended with a 9-fold volume of ion-exchanged water, left to stand until insoluble residues had settled down, and subjected to pH measurement with a pH meter (D-54, Horiba Co, Ltd., Kyoto, Japan).

### Measurement of short chain fatty acids in contents of the small intestine and contents of the cecum and colon

Concentrations of acetic acid and L-lactic acid in contents of the small intestine and contents of the cecum and colon were determined by enzymatic kits (Roche Diagnostics, Manheim, Germany).

### DNA extraction from intestinal contents

For DNA sequencing and quantitative real-time PCR, DNA extraction from intestinal contents was performed as previously described[26].

### Illumina library generation and DNA sequencing

16S rDNA analysis of the microbial community structure in intestinal contents was performed using a MiSeq (Illumina, San Diego, CA, USA). The V3-V4 region of 16S rDNA was amplified using a forward primer (5’-TCGTCGGCAGCGTCAGATGTGTATAAGAGACAGCCTACGGGNGGCWGCAG -3’) and a reverse primer (5’-GTCTCGTGGGCTCGGAGATGTGTATAAGAGACAGGACTACHVGGGTATCTAATCC -3’), which were ligated with overhang Illumina adapter consensus sequences. The initial PCR reaction was performed with the following program: 95°C for 3 min followed by 25 cycles consisting of 95°C for 30 sec, 55°C for 30 sec and 72°C for 30 sec. After 25 cycles, the reaction was completed with a final extension of 5 min at 72°C on a Veriti thermal cycler (Thermo Fisher Scientific, Waltham, MA, USA). The amplicon was purified using AMPure XP magnetic beads (Beckman Coulter, Brea CA, USA). The Illumina Nextera XT Index kit (Illumina) with dual 8-base indices was used to allow for multiplexing. PCR reactions were performed to incorporate two unique indices to the 16S amplicons. Cycling conditions consisted of 95°C for 3 min followed by eight cycles of 95°C for 30 sec, 55°C for 30 sec and 72°C for 30 sec, followed by a final extension cycle of 72°C for 5 min. After purification with AMPure XP beads, the purified barcoded library was fluorometrically quantified using a QuantIT PicoGreen ds DNA Assay Kit (Invitrogen Life Technologies). Libraries were then diluted to 4 nM using 10 mM Tris-HCl (pH 8.0), followed by pooling the same volume for multiplex sequencing. The multiplexed library pool (10 pM) was spiked with 12.5% PhiX control DNA (10 pM) to improve base calling during sequencing. Sequencing was conducted using a 2×250-bp paired-end run on a MiSeq platform with MiSeq Reagent Kit v2 chemistry (Illumina). The nucleotide sequence dataset was deposited in the Sequence Read Archive of the DNA Data Bank of Japan (DDBJ) under the accession number DRA005531.

### DNA sequence analysis

Demultiplexing and removal of indices were performed using the MiSeq Reporter software (Illumina). Subsequently, sequence files were exported from the MiSeq Reporter software for further steps. Quality filtering (QV ≥ 30) and merging of paired reads to be joined together were conducted using CLC Genomics Workbench (CLC Bio, Qiagen, Aarhus, Denmark). The determined 16S rDNA sequences were subjected to homology searching using local_RDP_classifier (World fusion, Tokyo, Japan). Obtained sequences were assigned to genus levels with 50% confidence threshold using Metagenome@KIN software (World fusion).

### Statistical analysis

Data are shown as means ± standard error (S.E.) except when median and interquartile range is indicated. Statistical significance was set at *p*<0.05. For comparison between two groups, significance was tested by the Mann-Whitney *U* test when variances were unequal. For multiple comparisons, significance was tested by the Dunnett test or Tukey-Kramer test when variances were equal and by the Steel test or Steel Dwass test when variances were unequal.

## Results

### Prophylactic oral administration of BBG9-1 reduces diarrhea in RV-infected mice

**Fig 1:**
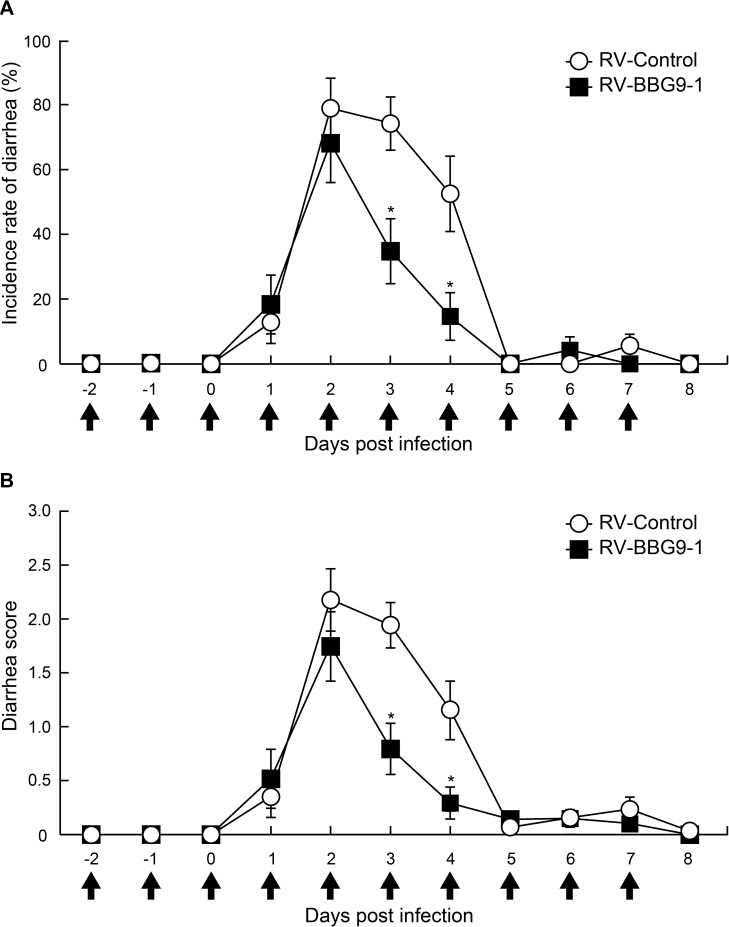
Beneficial effects of prophylactic oral administration of BBG9-1 on RV-induced diarrhea in mice. Mice were orally administered PBS or BBG9-1 (3.0 ×10^7^ CFU/day/mice) once daily for 10 days from 2 days before to 7 days after RV infection including the day of RV infection (arrows). Mice were orally inoculated with 50 μl of cell-cultured supernatant containing 1.5 ×10^6^ PFU of SA11. (A) Incidence rate of diarrhea of mice was monitored every day. Data from three independent experiments are shown as means ± S.E. (n = 6 litters for each group). *p < 0.05 by the Mann-Whitney U test. (B) Diarrhea score of each mouse was monitored daily for severity of diarrhea index as defined in the Materials and Methods section. Data from three independent experiments are shown as means ± S.E. (n = 6 litters for each group). *p < 0.05 by the Mann-Whitney U test.

We examined the prophylactic effects of orally administered BBG9-1 on incidence rates of diarrhea and diarrhea scores in RV-infected mice. In mice in the RV-Control group, incidence rates of diarrhea were 74.2 ± 8.2% at 3 dpi and 52.5 ± 11.7% at 4 dpi, whereas incidence rates of diarrhea in mice in the RV-BBG9-1 group were significantly decreased to 34.8 ± 10.0% at 3 dpi and 14.6 ± 7.4% at 4 dpi (Fig 1A). Diarrhea scores in the RV-Control group were 1.9 ± 0.2 at 3 dpi and 1.2 ± 0.3 at 4 dpi, whereas those in the RV-BBG9-1 group were significantly decreased to 0.8 ± 0.2 at 3 dpi and 0.3 ± 0.1 at 4 dpi (Fig 1B). Thus, oral administration of BBG9-1 from 2 days before RV infection had a prophylactic effect on RV-induced diarrhea.

### Therapeutic oral administration of BBG9-1 reduces diarrhea in RV-infected mice

**Fig 2:**
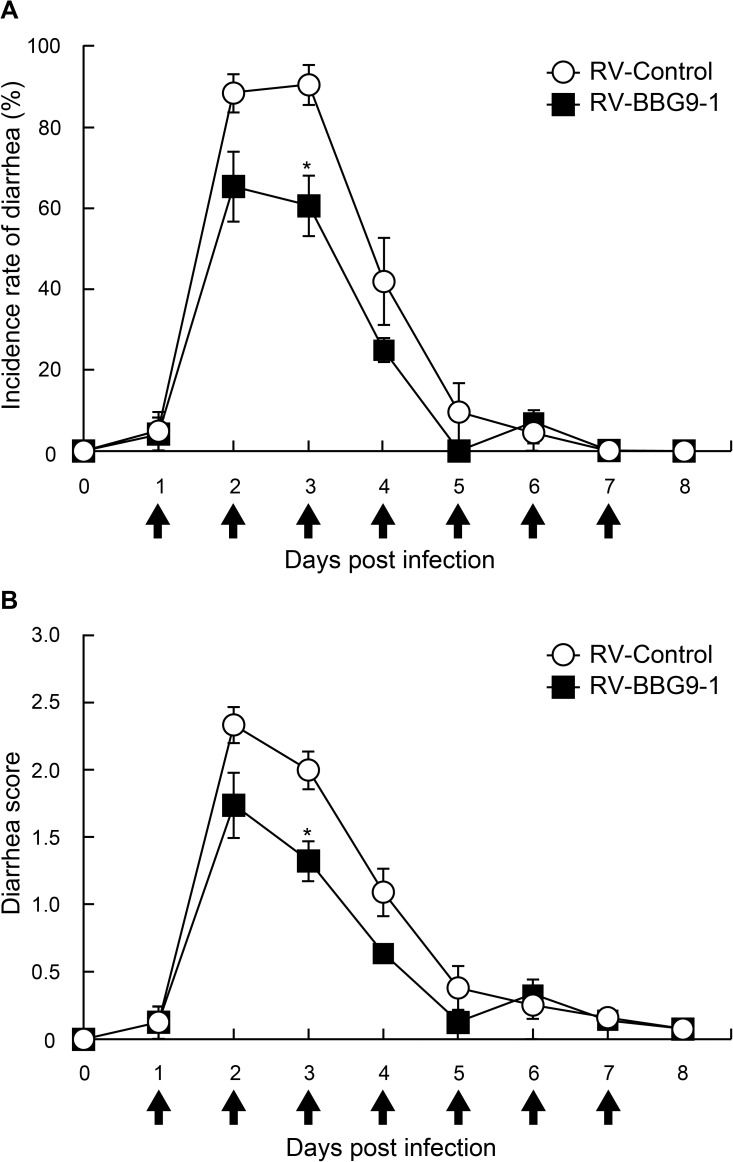
Beneficial effects of therapeutic oral administration of BBG9-1 on RV-induced diarrhea in mice. Mice were orally administered PBS or BBG9-1 (3.0 ×10^7^ CFU/day/mice) once daily for 7 days from 1 to 7 days after RV infection (arrows). Mice were orally inoculated with 50 μl of cell-cultured supernatant containing 1.5 ×10^6^ PFU of SA11. (A) Incidence rate of diarrhea of mice was monitored every day. Data from two independent experiments are shown as means ± S.E. (n = 6 litters for each group). *p < 0.05 by the Mann-Whitney *U* test. (B) Diarrhea score of each mouse was monitored daily for severity of diarrhea index as defined in the Materials and Methods section. Data from independent experiments are shown as means ± S.E. (n = 6 litters for each group). *p < 0.05 by the Mann-Whitney *U* test.

We examined the therapeutic effects of BBG9-1 on incidence rates of diarrhea and diarrhea scores in RV-infected mice by means of oral administration of BBG9-1 from 24 hr post infection, when initial shedding of RV has already started[19]. Incidence rates of diarrhea in the RV-Control group were 88.4 ± 4.7% at 2 dpi and 90.5 ± 5.0% at 3 dpi. In contrast, those in the RV-BBG9-1 group were decreased significantly to 65.3 ± 8.7% at 2 dpi and 60.5 ± 7.5% at 3 dpi (Fig 2A). Similarly, diarrhea scores in the RV-Control group were 2.0 ± 0.1 at 3 dpi and 1.1 ± 0.2 at 4 dpi, whereas those in the RV-BBG9-1 group were significantly decreased to 1.3 ± 0.1 at 3 dpi and 0.6 ± 0.1 at 4 dpi (Fig 2B). The dose-response effect of orally administered BBG9-1 was also confirmed in an experiment in which BBG9-1 was orally administered at three doses of 3.0 × 10^6^, 3.0 × 10^7^, and 3.0 × 10^8^ CFU/mouse/ day (S1 Fig). Thus, oral administration of BBG9-1 from 1 dpi was shown to have a therapeutic effect on RV-induced diarrhea.

### Therapeutic oral administration of BBG9-1 alleviates RV-induced damage in the small intestine

**Fig 3:**
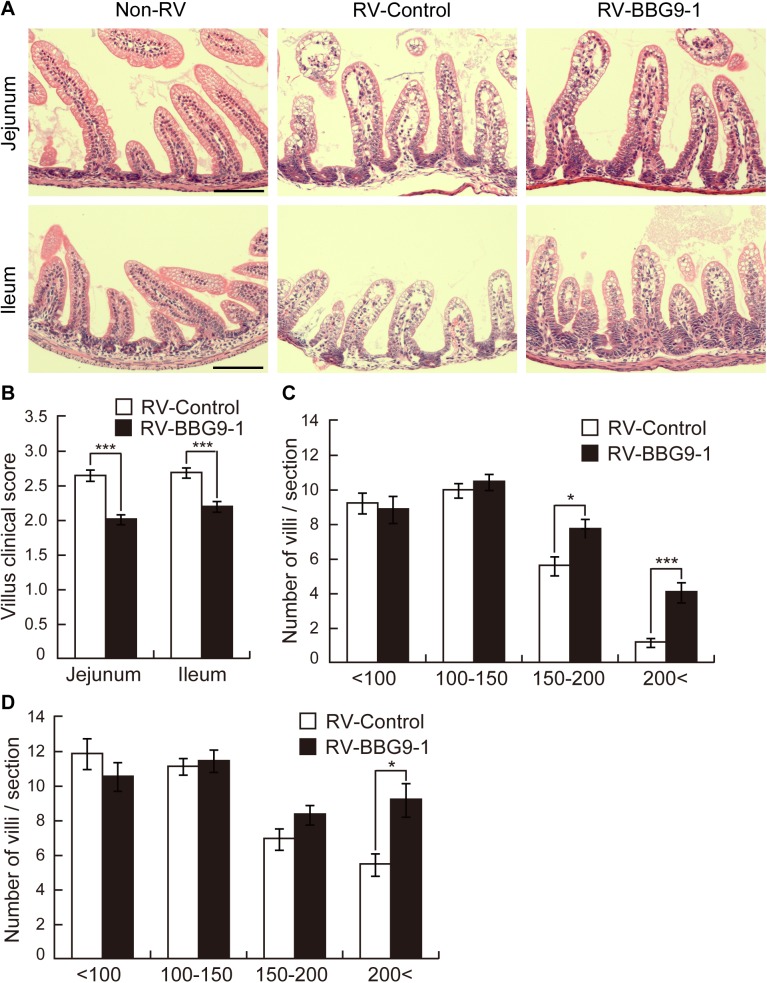
Therapeutic oral administration of BBG9-1 alleviates histopathologic damage of the small intestine of RV-infected mice. Oral administration of BBG9-1 and inoculation of RV were performed as described in the legend of Fig 2. Small intestines were removed 2 days after infection. Paraffin-embedded sections were stained with HE reagent. (A) Upper and lower panels show HE-stained sections of the jejunum and ileum, respectively. Scale bar represents 100 μm. (B) Clinical scores of jejunal and ileal villi were evaluated as defined in the Materials and Methods section. Data are shown as means ± S.E. (n = 191–263 villi for each group). ***p < 0.001 by the Mann-Whitney *U* test. Villus lengths in (C) the jejunum and (D) the ileum were measured and classified into four groups as defined in the Materials and Methods section. Data are shown as means ± S.E. (n = 33–43 sections for each group). *p < 0.05; ***p < 0.001 by the Mann-Whitney *U* test.

RV infects villus enterocytes of the small intestine and causes direct damage to villi including epithelial vacuolization and villus shortening, which results in a decrease in disaccharidases and malabsorption of nutrients and water, leading to severe diarrhea[25,27]. In order to examine the histopathological effects of orally administrated BBG9-1, we determined histopathological scores and lengths of the jejunal and ileal villi by H&E staining. Epithelial vacuolization, breakage of epithelial barrier function and villous disruption were not observed in the Non-RV group but were observed in both the RV-Control and RV-BBG9-1 group, but villi in the RV-BBG9-1 group appeared to be less damaged than those in the RV-Control group (Fig 3A). Corresponding to these observations, clinical scores of villi from the RV-Control group were 2.6 ± 0.1 in the jejunum and 2.7 ± 0.1 in the ileum, whereas those from the RV-BBG9-1 group were significantly reduced to 2.0 ± 0.1 in the jejunum and 2.2 ± 0.1 in the ileum (Fig 3B). In the jejunum, the number of villi with lengths of < 200 μm in the RV-BBG9-1 group was significantly larger (1.7-times larger) than that in the RV-Control. In the ileum, the numbers of villi with lengths of 150–200 μm and < 200 μm in the RV-BBG9-1 group were significantly larger (1.4-times and 3.5-times larger, respectively) than those in the RV-Control (Fig 3C). Taken together, these results indicate that therapeutic oral administration of BBG9-1 abates the characteristic lesions of small intestinal villi after RV infection.

### Therapeutic oral administration of BBG9-1 suppresses RV replication in the intestine of RV-infected mice

**Fig 4:**
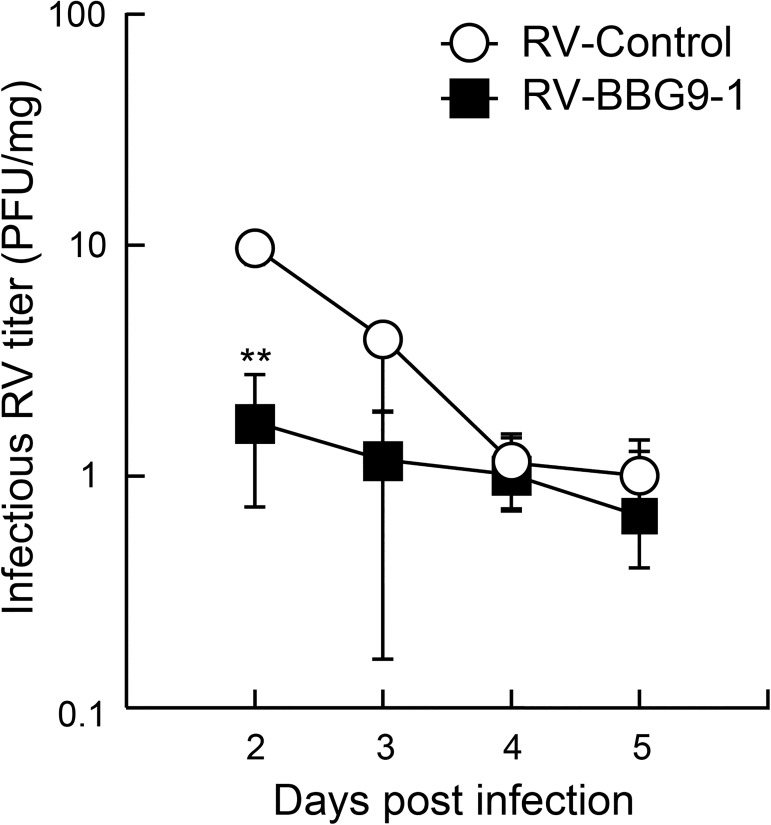
Therapeutic oral administration of BBG9-1 suppresses RV replication in the intestine of RV-infected mice. Oral administration of BBG9-1 and inoculation of RV were performed as described in the legend of Fig 2. Virus titers (PFU/mg) in mixtures of cecum and colonic contents were measured by plaque assays 2–5 days after infection. Data are shown as medians and interquartile range (n = 5 or 6 pups for each group). **p < 0.01 by the Mann-Whitney U test.

To determine whether oral administration of BBG9-1 affects viral replication in the intestine of RV-infected mice, we measured virus titers in mixtures of cecum and colonic contents from suckling mice by a plaque-forming assay. The median virus titer in the RV-Control group at 2 dpi was 9.5 PFU/mg, whereas that in the RV-BBG9-1 group was significantly decreased to 1.7 PFU/mg (Fig 4). These results indicate that therapeutic oral administration of BBG9-1 also suppresses RV proliferation in the intestine of RV-infected mice.

### Therapeutic oral administration of BBG9-1 alleviates RV-induced loss of acidic mucin-positive goblet cells in the small intestine

Goblet cells are characteristic cells of the intestinal epithelium and they secrete several mucosal protective factors. Mucins are high-molecular-mass glycoproteins present in the mucus coating the epithelial surfaces of the gastrointestinal tract, and they neutralize rotavirus infection through direct interaction with the virus[28].

**Fig 5:**
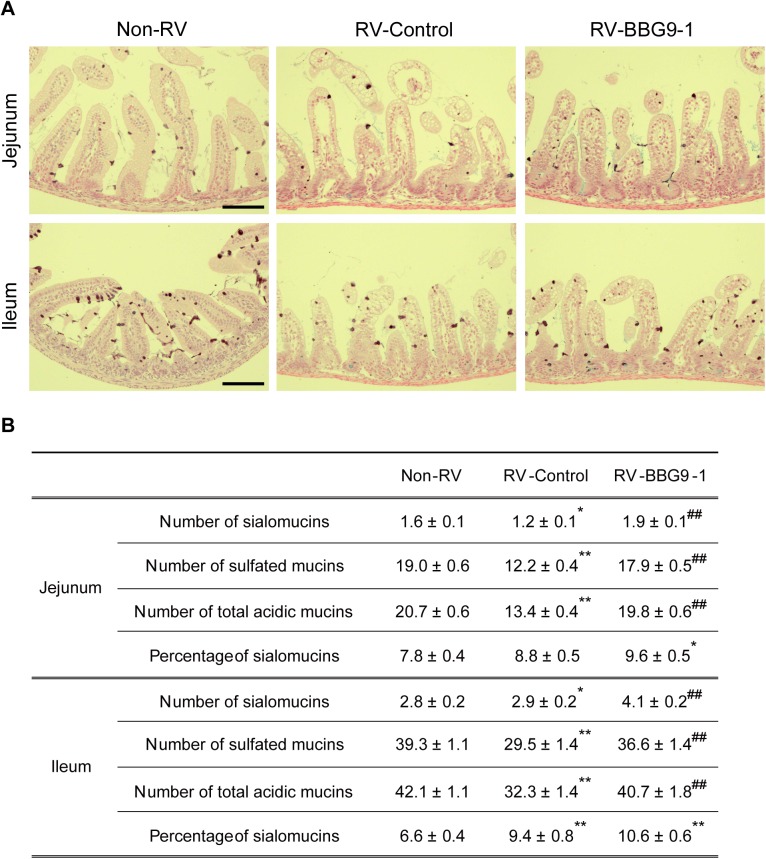
Therapeutic oral administration of BBG9-1 alleviates RV-induced loss of acidic mucin in the small intestine. Oral administration of BBG9-1 and inoculation of RV were performed as described in the legend of Fig 2. Small intestines were harvested 2 days after infection. Paraffin-embedded sections were stained with HID/AB. (A) Upper and lower panels show HID/AB-stained sections of the jejunum and ileum, respectively. Scale bar represents 100 μm. HID-positive brown and AB-positive blue were defined as goblet cells containing sulfated mucins and sialomucins, respectively. (B)The number of goblet cells containing each mucin was calculated per length of villi, and the proportions of goblet cells containing sialomucins in the jejunum and ileum were calculated. Data are shown as means ± S.E. (n = 33–43 sections for each group). *p < 0.05 and **p<0.01 compared with the Non-RV group; ^##^*p*<0.01 compared with the RV-Control group by the Tukey-Kramer test.

We performed a pathological examination to determine whether therapeutic oral administration of BBG9-1 affects both the quality and quantity of mucins. Since goblet cells have been reported to contain abundant acidic mucins after RV infection[29], we performed HID-AB staining to determine whether acidic mucin is a sulfated form or non-sulfated form, sialomucin. In the jejunum, the numbers of goblet cells containing sialomucins, sulfated mucins and total acidic mucins in the RV-Control group were significantly smaller than those in the Non-RV group by 27.5%, 35.9% and 35.3%, respectively. Interestingly, oral administration of BBG9-1 significantly abated RV-induced loss of jejunal goblet cells containing sialomucins, sulfated mucins and total acidic mucins to degrees comparable to those in the Non-RV group. Similarly, in the ileum, RV infection significantly decreased the numbers of goblet cells containing sulfated mucins and total acidic mucins by 25.0% and 23.1%, respectively, whereas oral administration of BBG9-1 alleviated RV-induced loss of goblet cells containing sulfated mucins and total acidic mucins to non-RV infection levels. In addition, a significant increase (1.5 times) in the number of sialomucin-positive goblet cells was observed in the ileum from the RV-BBG9-1 group compared with that from the Non-RV group (Fig 5A and 5B). These results indicate that retention of the number of acidic mucin-positive goblet cells at a normal level contributes to the therapeutic effects of orally administered BBG9-1 against RV gastroenteritis.

### Therapeutic oral administration of BBG9-1 affects gene expression in the small intestine of RV-infected mice

**Fig 6:**
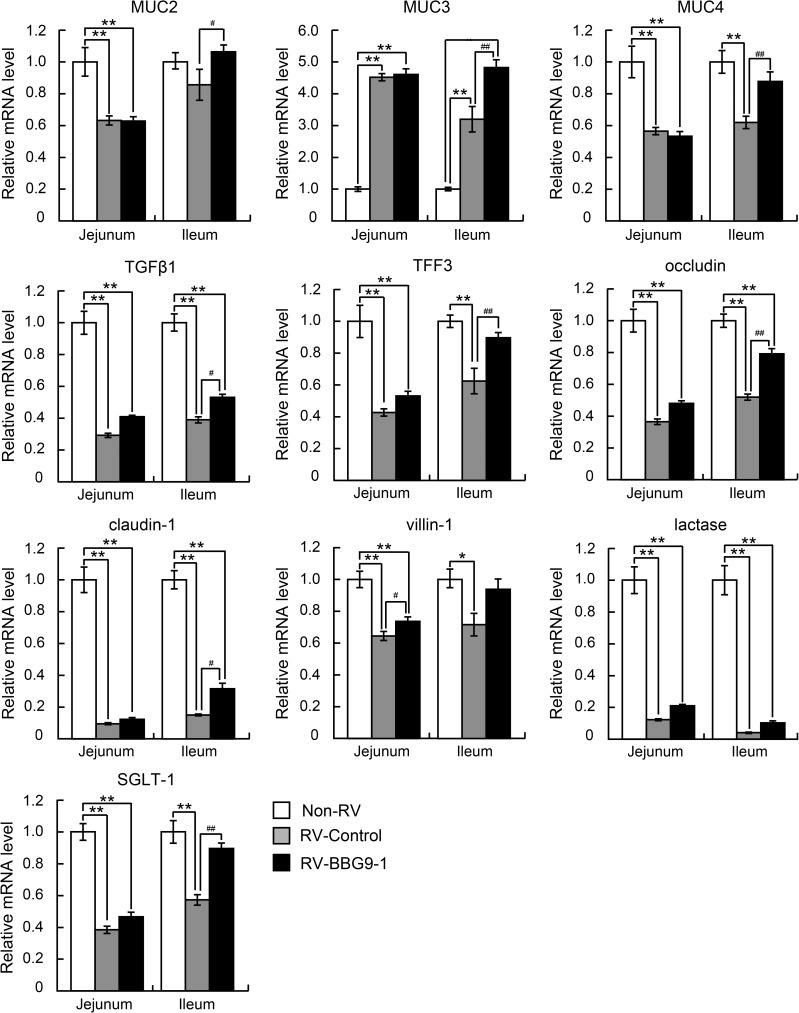
Therapeutic oral administration of BBG9-1 affects gene expression in the small intestine of RV-infected mice. Oral administration of BBG9-1 and inoculation of RV were performed as described in the legend of Fig 2. Small intestines were harvested 2 days after infection. GAPDH was used for normalizing each gene expression. The mRNA levels of each gene in the RV-Control group and the RV-BBG9-1 group are shown as relative values to those in the Non-RV group. Data are shown as means ± S.E. (n = 6 pups for each group). *p < 0.05 and **p < 0.01 compared with the Non-RV group; ^#^*p*<0.05 and ^##^*p*<0.01 compared with the RV-Control group by the Tukey-Kramer test.

To further examine the precise mechanisms underlying the therapeutic effects of orally administered BBG9-1 against RV gastroenteritis, we analyzed jejunal and ileal mRNA levels of mucosal protective factors (MUC2, MUC3, MUC4, TGFβ1 and TFF3), components of tight junctions (occludin and claudin-1), and factors implicated in maintaining structures of brush borders (villin-1), nutritional absorption (lactase) and sodium absorption (SGLT-1). The mRNA levels of all of the target genes in both the jejunum and ileum in the RV-Control group were markedly decreased compared with those in the Non-RV group, except for remarkable increases in the mRNA levels of MUC3 in both the jejunum and ileum. In contrast, mRNA level of villin-1 in the jejunum in the RV-BBG9-1 group and mRNA levels of MUC2, MUC3, MUC4, TGFβ1, TFF3, occludin, claudin-1 and SGLT-1 in the ileum in the RV-BBG9-1 group were significantly higher than those in the RV-Control group; villin-1, by 1.2 times; MUC2, by 1.3 times; MUC3, by 1.6 times; MUC4, by 1.4 times; occludin, by 1.5 times; TGFβ1, by 1.4 times; TFF3, by 1.4 times; claudin-1 by 2.1 times; SGLT-1, by 1.6 times (Fig 6). Considering these results, upregulation of MUC family members contributes to the retention of acidic mucins to a non-infection level. This result was effectively collaborated with other mucosal protective factors, contributing to the preservation of intestinal functions such as barrier function and sodium absorption.

### Therapeutic oral administration of BBG9-1 affects the intestinal environment of RV-infected mice

**Fig 7:**
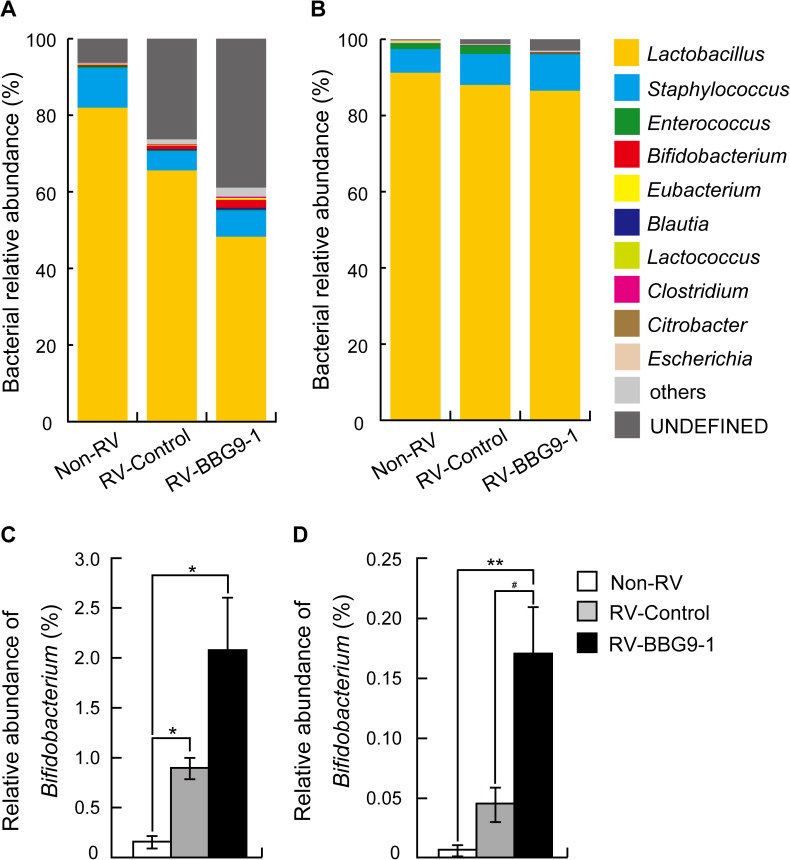
Both RV infection and therapeutic oral administration of BBG9-1 affect the gut microbiota of mice. Oral administration of BBG9-1 and inoculation of RV were performed as described in the legend of Fig 2. Contents of the small intestine and contents of the cecum and colon were harvested 2 days after infection and subjected to microbiota analysis. Bacterial relative abundances in (A) the small intestine and (B) the cecum and colon were measured. Data are shown as means (n = 5–8 pups for each group). Bifidobacterium relative abundances in (C) the small intestine and (D) the cecum and colon were measured. Data are shown as means ± S.E (n = 5–8 pups for each group) **p < 0.01 compared with the Non-RV group; ^#^*p*<0.05 compared with the RV-Control group by the Tukey-Kramer test.

Finally, we investigated the effects of orally administered BBG9-1 on the intestinal environment including the amounts of short chain fatty acids, pH and constitution of the microbiota. In the RV-Control group, the levels of both acetic acid and L-lactic acid in small intestinal contents were significantly higher (1.4 times and 1.5 times, respectively) than those in the Non-RV group, implying that undigested lactose induces abnormal fermentation in the small intestine after RV infection. Meanwhile, oral administration of BBG9-1 tended to reduce the RV-induced increase in L-lactic acid in the small intestine by 20.0%. In the RV-Control group, the level of L-lactic acid in cecum and colonic contents was significantly higher (2.4 times) than that in the Non-RV group, while the level of acetic acid was equivalent to that in the Non-RV group. Oral administration of BBG9-1 significantly increased the amount of L-lactic acid (by 3.7 times) compared with that in the RV-Control group. Regarding pH, RV infection significantly shifted small intestinal contents to alkaline pH and tended to shift cecum and colonic contents to alkaline pH (*p* = 0.11 compared with the Non-RV group). Oral administration of BBG9-1 tended to normalize pH in cecum and colonic contents (*p* = 0.98 compared with the Non-RV group, Table 1). In the small intestine, microbiota analysis showed a decrease in *Lactobacillus* and increases in both *Bifidobacterium* and an undefined group in the RV-Control group. Decreased *Lactobacillus* and increased *Bifidobacterium* and an undefined group were more clearly observed in the small intestine from the RV-BBG9-1 group than in the small intestine from the RV-Control group. In the cecum and colon, RV infection had little effect on the constitution of microbiota, while oral administration of BBG9-1 significantly increased *Bifidobacterium* (Fig 7A–7D). Considering that BBG9-1 has endogenous lactase activity, these results suggest that orally administered BBG9-1 by itself metabolizes undigested lactose to short chain fatty acids, resulting in normalization of alkaline pH in the cecum and colon.

**Table 1:** Effects of therapeutic oral administration of BBG9-1 on concentrations of acetic acid and L-lactic acid and pH in contents of the small intestine and large intestine.

		Non-RV	RV-Control	RV-BBG9-1
Small intestinal contents	Acetic acid (mg/mL)	178 ± 13	251 ± 7**	240 ± 11**
	L-lactic acid (mg/mL)	433 ± 34	651 ± 36*	512 ± 68
	pH	6.19 ± 0.03	6.40 ± 0.04**	6.43 ± 0.02**
Cecum and colonic contents	Acetic acid (mg/mL)	309 ± 57	317 ± 38	399 ± 31
	L-lactic acid (mg/mL)	344 ± 70	841 ± 55*	1262 ± 155**^,^ ^#^
	pH	5.75 ± 0.08	6.23 ± 0.15	5.79 ± 0.21

Oral administration of BBG9-1 and inoculation of RV were performed as described in the legend of Fig 2. Contents of the small intestine and contents of the cecum and colon were harvested 2 days after infection. Data are shown as means ± S.E. (n = 6 pups for each group).

*p < 0.05.

**p < 0.01 compared with the Non-RV group.

^#^*p*<0.05 with the RV-Control group by the Tukey-Kramer test or Steel Dwass test.

## Discussion

**Fig 8:**
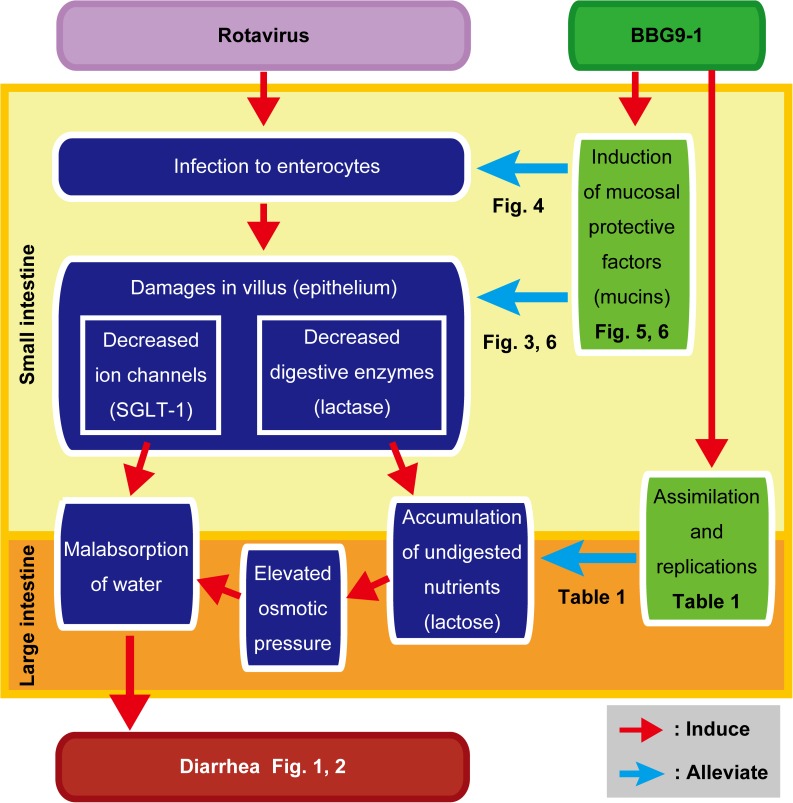
A model for the palliative effect of oral administration of BBG9-1 against RV gastroenteritis. Oral administration of BBG9-1 induced mucosal protective factors, such as mucin, TFF3 and TGFβ1. Acidic mucins neutralize RV infection and replication in the small intestine. Mucosal protective factors coordinately protect against damage after RV infection to alleviate decreased gene expression of SGLT-1, which plays a pivotal role in water absorption. Orally administered BBG9-1 replicates in the large intestine to assimilate undigested nutrients, contributing to reduction in osmotic pressure. Improvement in water malabsorption in both the small and large intestines contributes to alleviation of RV-induced diarrhea.

In the present study, we clearly showed that both prophylactic and therapeutic oral administrations of BBG9-1 palliate RV-induced diarrhea (Figs 1 and 2). Furthermore, we obtained findings supporting that oral administration of BBG9-1 alleviates RV-induced diarrhea through induction of mucosal protective factors, which neutralize RV replication and prevent the development of RV-induced lesions. In addition to the effects on the small intestine, orally administered BBG9-1 was shown to replicate in the large intestine to assimilate undigested nutrients, which could contribute to the alleviation of osmotic diarrhea (Fig 8).

In addition, we found that oral administration of BBG9-1 significantly increases gene expression of MUC2, MUC3 and MUC4 in the ileum. Among them, the mRNA level of MUC3 was markedly increased after RV infection (Fig 6), possibly as a result of the action of the host defense system against RV. Induction of MUC3 has been shown to be involved in suppression of *Escherichia coli* adherence to intestinal epithelia by *Lactobacillus rhamnosus* GG and *Lactobacillus plantarum*[30]. MUC3 has been reported to be expressed in two forms: a full-length membrane-associated form found in columnar cells and a carboxyl-cleaved soluble form found in goblet cells[31]. Since columnar cells play an important role in nutrient absorption by bearing microvilli in which channels and brush border enzymes including lactase are highly enriched, the robustly expressed MUC3 could contribute to the preservation of microvillus functions such as digestion and absorption, leading to alleviation of osmotic diarrhea.

The influence of RV infection on the characteristics of mucins has been reported. Boshuizen *et al*. showed that the RV infection increases the proportion of sialomucins[29], being consistent with our results (Fig 5). They also showed that mucins derived from RV-infected mice have more protective capacity than do those derived from non-RV infected mice, raising the possibility that sialomucins are more effective in neutralizing RV. In support of this idea, RV has been reported to interact with sialic acid residues in molecules including mucins[32,33]. Interestingly, it has been reported that osteoponcin, which is found in sialated forms in breast milk[34,35], contributes to host responses against RV infection[36,37]. These studies indicate the possibility that an increased proportion of sialated mucin by oral administration of BBG9-1 serves for effective protection against RV infection.

Since epithelia are exposed to several environmental stresses including pathogens, both regulation of intestinal permeability and efficient repair of the intestinal epithelium are essential to maintain the integrity of the epithelial barrier. RV infection impairs intestinal barrier function, which is shown as decreases in the gene expression of tight junction components including occludin and claudin-1[38]. In the present study, we found that administered BBG9-1 rescues decreased mRNA levels of occludin and claudin-1 after RV infection (Fig 6). We also found that the culture supernatant of BBG9-1 improves the impaired transendothelial electrical resistance (TER) of human colonic epithelial Caco-2 cells after co-culture with macrophage-like THP-1 cells (S1 Table). These findings suggest that oral administration of BBG9-1 consolidates tight junctions, thereby conferring resistance against RV-induced damage. Several processes, including reconstruction of the mucosa and proliferation and differentiation of epithelial cells, are involved in the restoration of mucosal functions. Both TGFβ1 and TFF3 have been reported to play important roles in protecting intestinal mucosa and prompting reconstruction of damaged mucosa[39]. Viliin, which is an actin-binding protein expressed in microvilli of the intestine, has also been proposed to be involved in colonic wound repair by remodeling of actin filaments in microvilli[40,41]. Moreover, the cysteine-rich domain of MUC3 has been shown to promote cell migration and improve wound healing of the colonic mucosa[42]. Therefore, up-regulation of MUC3, TGFβ1, TFF3 and villin-1 by administration of administrated BBG9-1 might coordinately contribute to the repair of epithelial damage after RV infection (Fig 6). Taken together, the results raise the possibility that the protective effect of orally administered BBG9-1 arises from enhanced tight junctions and epithelial repairing.

Approximately 80% of water is absorbed in the small intestine. The net movement of water across the cell membrane is coupled with absorption of solutes, particularly sodium. SGLT-1 is mainly expressed in the brush border membrane of the intestine, and its contribution to Na^+^-D-glucose cotransport across the brush border membrane has been proposed[43,44]. In the present study, we found that oral administration of BBG9-1 rescued the decreased mRNA level of SGLT-1 in the ileum after RV infection (Fig 6), suggesting that improved water absorption in the small intestine contributes to palliation of diarrhea.

Close relationships between intestinal microbiota and occurrence of diarrhea have been proposed[45,46,47]. In the present study, RV infection caused microbiota alteration and abnormal fermentation shown as significant increases in both acetic acid and L-lactic acid. In contrast, orally administered BBG9-1 had little effect on microbiota in the small intestine, except for an increase in an undefined group (Fig 7). This increase in an undefined group may be due to a poor understanding of the infantile microbiota, especially in the small intestine. Since the infantile microbiota plays an important role in the formation of a basis for intestinal functions including host defense programs, further studies are required for examining how orally administered BBG9-1 influences the microbiota of the small intestine and what its effect is on host defense function after RV infection.

Probiotics stimulate cells in gut-associated lymphoid tissue (GALT) such as Peyer’s patch cells to activate defense lines against several pathogens[48]. The involvement of IgA, IgG, IgM and IFNγ in alleviation of RV-induced diarrhea was previously reported[17,18,19,20]. Since intestinal structures including GALT are immature in a suckling mouse[49], effective activation of gut immunity is required for continuous stimuli by an antigen. Therefore, prolonged or multiple administrations of probiotics might be needed for acquiring anti-RV effects of probiotics. Actually, Zhang *et al*. achieved a therapeutic effect of *Lactobacillus rhamnosus* GG against RV gastroenteritis by daily multiple administrations[20]. However, the present study showed that BBG9-1 had a therapeutic effect with single-dose administration. This fast response of BBG9-1 may be consistent with our proposed model that orally administered BBG9-1 exerts palliative effects through not augmentation of host immunity but induction of mucosal protective factors. Actually, IFNγ was not detected at the protein level in plasma harvested at 2 dpi in the present study.

Considering the rapid therapeutic effects of oral administration of BBG9-1 against RV gastroenteritis including decreased infectious RV titers, a more direct effect of BBG9-1 to inhibit RV replication might be involved in the anti-RV mechanism of BBG9-1. Suppressive effects on RV replication were reported for probiotics including *Bifidobacterium thermophilum*, *Bifidobacterium longum* and *Lactobacillus acidophilus*[19,50,51]. Recently, Chenoll *et al*. showed that an 11-amino-acid peptide produced by *Bifidobacterium longum* subsp. *infantis* CECT 7210 has the ability to inhibit RV replication *in vitro*[52]. Further studies are needed to clarify other mechanisms underlying the palliative effect of orally administered BBG9-1 against RV gastroenteritis.

In conclusion, the present study showed that both prophylactic and therapeutic oral administrations of BBG9-1 palliate RV gastroenteritis partly through protection against RV-induced lesions by induction of mucosal protective factors. Therefore, both prophylactic and therapeutic administrations of BBG9-1 can be effective for the management of RV gastroenteritis. Further studies are required to determine whether oral administration of BBG9-1 has the same protective effect against RV infection in humans. Given that the anti-RV effect of BBG9-1 is partly through the protective effect of intestinal mucus, it is thought that oral administration of BBG9-1 would show similar protective effects against other types of infectious gastroenteritis, especially that caused by astroviruses and enteric adenoviruses that interact with sialic acids as RV does[32].

## Supporting information

S1 Fig Dose-response effect of oral administration of BBG9-1 on RV-induced diarrhea in mice. Oral administration of BBG9-1 (arrows) and inoculation of RV were performed as described in the legend of Fig 2. (A) Incidence rate of diarrhea of mice was monitored every day. Data from two independent experiments are shown as means ± S.E. (n = 6 litters for each group). *p < 0.05 and **p < 0.01 by the Dunnett test. (B) Diarrheal score of each mouse was monitored daily for severity of diarrhea index as defined in the Materials and Methods section. Data from two independent experiments are shown as means ± S.E. (n = 6 litters for each group). *p < 0.05 by the Steel test.
(TIF)

S1 File Supplemental procedures. (DOCX)

S1 Table Protective effect of BBG9-1 culture supernatant on epithelial barrier disruption in co-culture of Caco-2 cells with macrophage-like THP-1 cells. (DOCX)
