# Supplementary figures and images for: Oral administration of Bifidobacterium bifidum G9-1 alleviates rotavirus gastroenteritis through regulation of intestinal homeostasis by inducing mucosal protective factors
Source: PLoS One. 2017 Mar 27;12(3):e0173979. doi: 10.1371/journal.pone.0173979 (PMC5367788; doi:10.1371/journal.pone.0173979)

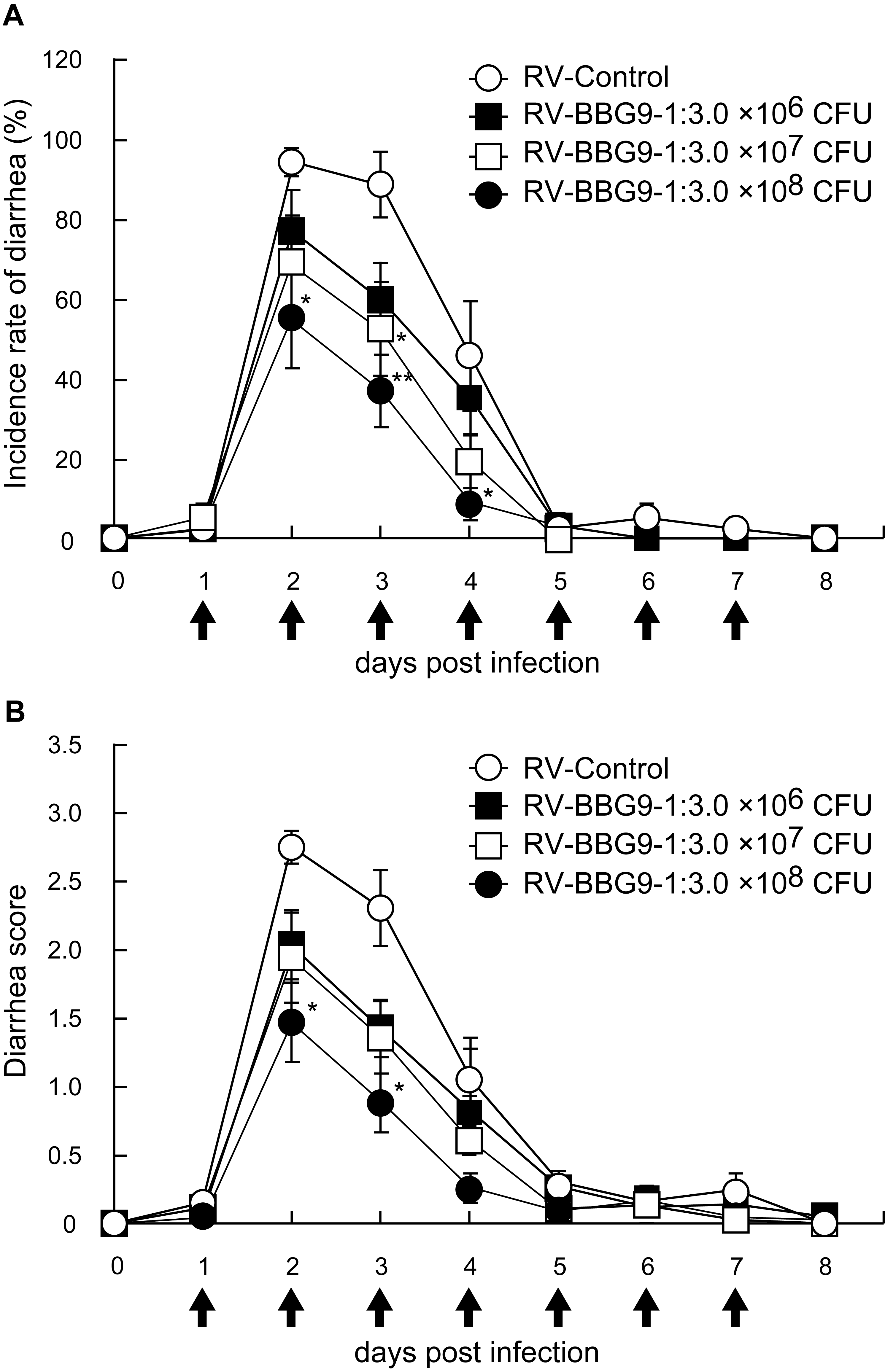

Supplement: S1 Fig — Oral administration of BBG9-1 (arrows) and inoculation of RV were performed as described in the legend of Fig 2. (A) Incidence rate of diarrhea of mice was monitored every day. Data from two independent experiments are shown as means ± S.E. (n = 6 litters for each group). *p < 0.05 and **p < 0.01 by the Dunnett test. (B) Diarrheal score of each mouse was monitored daily for severity of diarrhea index as defined in the Materials and Methods section. Data from two independent experiments are shown as means ± S.E. (n = 6 litters for each group). *p < 0.05 by the Steel test. (TIF) [file pone.0173979.s001.tif]
