# Supplementary material for: Oral administration of Bifidobacterium bifidum G9-1 alleviates rotavirus gastroenteritis through regulation of intestinal homeostasis by inducing mucosal protective factors
Source: PLoS One. 2017 Mar 27;12(3):e0173979. doi: 10.1371/journal.pone.0173979 (PMC5367788; doi:10.1371/journal.pone.0173979)
Supplement: S1 File — (DOCX) [file pone.0173979.s002.docx]

**Supplemental procedures**

**Dose-responsive effect of oral administration of BBG9-1 on RV-induced diarrhea**

Two-day-old suckling mice were randomly divided into a control group and three BBG9-1 dosage groups. Suckling mice were orally administered 50 µl of PBS or PBS containing 3.0 ×10^6^, 3.0 ×10^7^ or 3.0 ×10^8^ CFU of BBG9-1 for 7 days daily from 1 to 7 days after RV infection. Seven-day-old suckling mice were orally inoculated with 50 µl of cell-cultured supernatant containing 1.5 ×10^6^ plaque-forming units (PFU) of SA-11. Incidence rate of diarrhea and diarrhea score were measured as described in Materials and Methods.

**BBG9-1 culture supernatant**

BBG9-1 was cultured for 18 h at 37°C in GAM broth (Nissui Pharmaceutical Co., Ltd., Tokyo, Japan) supplemented with 0.7% glucose and 0.1% Tween-80. The bacteria were washed twice with phosphate buffered saline (PBS) and cultured in MEM (Sigma) for 20 h at 37°C. Before usage, the BBG9-1 culture supernatant was adjusted to pH 7.4 and filter-sterilized using a membrane filter with a 0.22-µm pore size (Millipore, Billerica, MA, USA).

**Cells**

Human intestinal Caco-2 cells (RIKEN Cell Bank, Tsukuba, Japan) were grown in MEM (Sigma) supplemented with 10% heat-inactivated FBS (Gibco), 1% non-essential amino acids, penicillin (50 U/ml) and streptomycin (50 µg/ml) (Gibco). Human monocytic THP-1 cells (RIKEN Cell Bank, Tsukuba, Japan) were grown in RPMI1640 (Sigma) supplemented with 10% heat-inactivated FBS (Gibco), penicillin (50 U/ml) and streptomycin (50 µg/ml) (Gibco). Caco-2 cells and THP-1 cells were incubated at 37°C under a humidified atmosphere of 5% CO_2_ in air.

**Co-culture experiments**

Caco-2 cells were cultured in 24-well cell culture inserts with a 0.4-µm pore size at a density of 2.0 × 10^4^ cells/insert. THP-1 cells were cultured in 24-well plates at a density of 1.5 × 10^5^ cells/well and differentiated to macrophage-like cells by treatment with 100 nM phorbolmyristrate acetate for 4 days. The semipermeable support membrane on which Caco-2 cell monolayers had been cultured for 14 days was placed on a 24-well plate preloaded with macrophage-like THP-1 cells. The BBG9-1 culture supernatant or GAM broth was added to cell culture inserts (BBG9-1 group and Control group, respectively). TER was measured with Millicell ERS-2 (Millipore) at 0 and 24 h after co-culture in accordance with the manufacturer’s instructions. TER values in the Control group and the BBG9-1 group are shown as relative values to that in the THP-1 untreated group (Normal group).
