# Supplementary material for: Oral administration of Bifidobacterium bifidum G9-1 alleviates rotavirus gastroenteritis through regulation of intestinal homeostasis by inducing mucosal protective factors
Source: PLoS One. 2017 Mar 27;12(3):e0173979. doi: 10.1371/journal.pone.0173979 (PMC5367788; doi:10.1371/journal.pone.0173979)
Supplement: S1 Table — (DOCX) [file pone.0173979.s003.docx]

|  | Normal (-THP-1) | Control (+THP-1) | BBG9-1 (+THP-1) |
| --- | --- | --- | --- |
| Relative TER value (%) | 100.00 ± 1.84 | 80.37 ± 1.81** | 98.66 ± 1.95^##^ |

TER values in the Control group and the BBG9-1 group are shown as relative values to that in the Normal group. Data are shown as means ± S.E. (n = 6). ***p* < 0.01 compared with the Normal group; ^##^*p*<0.01 compared with the Control group by the Tukey-Kramer test.
